# Editorial: Positive Psychology in Healthcare Professionals

**DOI:** 10.3389/fpsyg.2022.883603

**Published:** 2022-03-28

**Authors:** Li Liu, Hui Wu, Tao Sun

**Affiliations:** ^1^Department of Social Medicine, School of Health Management, China Medical University, Shenyang, China; ^2^Department of Health Policy and Management, School of Public Health, Hangzhou Normal University, Hangzhou, China

**Keywords:** positive psychology, healthcare professionals, occupational health psychology, organizational behavior, human resource management

Healthcare system is necessary to address major health problems, such as severe chronic diseases and the pandemic of new infectious diseases, and to improve human health and well-being. Healthcare professionals are obligated to adapt to continuous changes and challenges in health service delivery for ensuring the quality of health services (Byers, 2017; Ghahramani et al., 2021). As a result, the physical and mental burdens on healthcare professionals would be raised, resulting in a range of negative health and work outcomes (Deng et al., 2019; Hill et al., 2022). Thus, healthcare professionals need positive psychological resources to copy with the stressful situations in workplaces worldwide (Liu et al., 2012; Tian et al., 2020; Bazargan-Hejazi et al., 2021). Current positive psychology research mainly focuses on boosting the positive emotions and capabilities of health workers, as well as supportive interpersonal relationships and organizational environments *via* exploring and cultivating a variety of positive psychological resources (Bazargan-Hejazi et al., 2021; Kletter et al., 2021). In recent years, there is increasing evidence that positive psychological resources can promote physical and mental health, and stimulate positive work attitudes and behaviors among healthcare professionals (Liu et al., 2012; Tian et al., 2020; Yang et al., 2020; Yang and Wu, 2021). At the same time, the effectiveness of various positive psychological interventions has also been widely confirmed in the context of health services (Xu et al., 2016; Kloos et al., 2019; Luo et al., 2019; Guo et al., 2020; Kunzler et al., 2020).

This Research Topic collects five research articles that develop our understanding of the means that some specific positive psychological resources can improve work engagement and life satisfaction, as well as sleep quality and subjective health, and reduce psychological stress and the conflict between work and family among healthcare professionals. In medical students, occupation expectation, subjective well-being, and learning burnout are the outcomes of positive psychological resources. These findings can clarify how and why positive psychological resources work in these populations, and contribute to the development of new intervention targets for healthcare professionals. The articles draw on the work of five positive psychological factors including trait emotional intelligence, inquisitiveness, resilience, subjective well-being, and social support.

In Chinese nurses, Zhang S.-e et al. examined the associations of character strengths with psychological stress, sleep quality, and subjective health status. The results showed that Chinese nurses possessed high levels of character strengths, including caring, self-control, and inquisitiveness. The inquisitiveness of nurses was negatively associated with psychological stress, whereas showed positive associations with both sleep quality and subjective health. Furthermore, psychological stress partially mediated the associations of character strength-inquisitiveness with sleep quality and subjective health. This study made a contribution to the underlying mechanisms of character strengths for improving the physical and mental health of nurses. In a sample of Pakistani nurses from public sector hospitals, Gull et al. explored the relationship between paternalistic leadership, polychronicity, and life satisfaction, as well as the mediating roles of work-family conflict and family-work conflict in these variable relationships. The results of this study indicated that both paternalistic leadership and polychronicity were positively related to life satisfaction. Furthermore, work-family conflict showed a partial mediating role in the relationships of paternalistic leadership and polychronicity with life satisfaction. However, family-work conflict only partially mediated the association of paternalistic leadership with life satisfaction. This study expanded the Conservation of Resources theory by incorporating the effects of paternalistic leadership and polychronicity on life satisfaction *via* work-family conflict and family-work conflict in nurses. A study of Tesi discussed how trait emotional intelligence facilitates the work-related well-being of healthcare professionals. The results revealed that trait emotional intelligence was directly and indirectly related to burnout and work engagement, and the indirect paths linked by end-user job demands and coworker-related job resources, respectively. As an individual difference characterizing healthcare professions, emotional intelligence can be evaluated as a concern of the personnel selection process in healthcare organizations. Since the occupational well-being of healthcare professionals was predicted by their capabilities of identifying and managing emotions, as well as maintaining supportive relationships with patients and coworkers, it is necessary to implement targeted intervention for promoting emotional intelligence.

In Chinese medical students, Wu et al. reported a sequential mediation of resilience and subjective well-being in the relationship between empathy and occupation expectation. Based on the findings, intervention for strengthening resilience and improving subjective well-being could be effective to identify professional values, and ameliorate occupation expectation, as well as reduce turnover in medical students. In addition, Zhang J.-Y. et al. explored whether social support can help medical students cope with learning burnout. The results showed that subjective social support and the utilization of social support may independently play a protective role in reducing learning burnout. In view of the positive effect of social support on a variety of positive psychological resources, it will be an indispensable link in the positive psychology research among healthcare professionals in future.

In view of the findings of this Research Topic, the importance of identifying and cultivating positive psychological resources should be increasingly emphasized in healthcare professionals. In fact, much more needs to be done to address the physical and mental burdens of healthcare professionals from the perspective of positive psychology, which is essential for their own occupational quality of life, and better patient care and health. In future, more longitudinal studies should be conducted to shed light on the causal relationship of positive psychological resources with health and work outcomes among healthcare professionals. Also, strengthening the positive psychology intervention among healthcare professionals is a beneficial choice for the future reform of occupational health management in hospitals.

## Author Contributions

LL drafted the editorial. HW and TS provided opinions and suggestions on the draft. LL, HW, and TS have made a substantial and intellectual contribution to the Research Topic and have approved the editorial for publication. All authors contributed to the article and approved the submitted version.

## Conflict of Interest

The authors declare that the research was conducted in the absence of any commercial or financial relationships that could be construed as a potential conflict of interest.

## Publisher's Note

All claims expressed in this article are solely those of the authors and do not necessarily represent those of their affiliated organizations, or those of the publisher, the editors and the reviewers. Any product that may be evaluated in this article, or claim that may be made by its manufacturer, is not guaranteed or endorsed by the publisher.

## References

[B1] Bazargan-Hejazi S. Shirazi A. Wang A. Shlobin N. A. Karunungan K. Shulman J.. (2021). Contribution of a positive psychology-based conceptual framework in reducing physician burnout and improving well-being: a systematic review. BMC Med. Educ. 21:593. 10.1186/s12909-021-03021-y 34823509 PMC8620251

[B2] Byers V. (2017). The challenges of leading change in health-care delivery from the front-line. J. Nurs. Manag. 25, 449–456. 10.1111/jonm.12342 26648566

[B3] Deng J. Sun Y. Lei R. Guo Y. Liu J. Yang T. (2019). Status of healthcare workers after comprehensive reform of urban public hospitals in Beijing, China: sustainable supply, psychological perception, and work outcomes. Hum. Resour. Health 17:77. 10.1186/s12960-019-0421-1 31660985 PMC6819331

[B4] Ghahramani S. Lankarani K. B. Yousefi M. Heydari K. Shahabi S. Azmand S. (2021). A systematic review and meta-analysis of burnout among healthcare workers during COVID-19. Front. Psychiatry 12:758849. 10.3389/fpsyt.2021.758849 34858231 PMC8631719

[B5] Guo Y. F. Lam L. Plummer V. Cross W. Zhang J. P. (2020). A WeChat-based “Three Good Things” positive psychotherapy for the improvement of job performance self-efficacy in nurses with burnout symptoms: a randomized controlled trial. J. Nurs. Manag. 28, 480–487. 10.1111/jonm.12927 31811737

[B6] Hill J. E. Harris C. Danielle L. C. Boland P. Doherty A. J. Benedetto V.. (2022). The prevalence of mental health conditions in healthcare workers during after a pandemic: systematic review meta-analysis. J. Adv. Nurs. 10.1111/jan.15175. [Epub ahead of print]. 35150151 PMC9111784

[B7] Kletter M. Harris B. Brown C. (2021). Outcomes, mechanisms and contextual factors of positive psychology interventions for health workers: a systematic review of global evidence. Hum. Resour. Health 27, 19–24. 10.1186/s12960-021-00564-5 33639979 PMC7910793

[B8] Kloos N. Drossaert C. H. C. Bohlmeijer E. T. Westerhof G. J. (2019). Online positive psychology intervention for nursing home staff: a cluster-randomized controlled feasibility trial of effectiveness acceptability. Int. J. Nurs. Stud. 98, 48–56. 10.1016/j.ijnurstu.2019.06.004 31295708

[B9] Kunzler A. M. Helmreich I. Chmitorz A. König J. Binder H. Wessa M.. (2020). Psychological interventions to foster resilience in healthcare professionals. Cochrane Database Syst. Rev. 7:CD012527. 10.1002/14651858.CD012527.pub2 32627860 PMC8121081

[B10] Liu L. Chang Y. Fu J. Wang J. Wang L. (2012). The mediating role of psychological capital on the association between occupational stress and depressive symptoms among Chinese physicians: a cross-sectional study. BMC Public Health 12:219. 10.1186/1471-2458-12-219 22436106 PMC3410796

[B11] Luo Y. H. Li H. Plummer V. Cross W. M. Lam L. Guo Y. F.. (2019). An evaluation of a positive psychological intervention to reduce burnout among nurses. Arch. Psychiatr. Nurs. 33, 186–191. 10.1016/j.apnu.2019.08.004 31753226

[B12] Tian F. Shu Q. Cui Q. Wang L. Liu C. Wu H. (2020). The mediating role of psychological capital in the relationship between occupational stress and fatigue: a cross-sectional study among 1,104 Chinese physicians. Front. Public Health 8:12. 10.3389/fpubh.2020.00012 32185156 PMC7058796

[B13] Xu X. Hu M. L. Song Y. Lu Z. X. Chen Y. Q. Wu D. X.. (2016). Effect of positive psychological intervention on posttraumatic growth among primary healthcare workers in China: a preliminary prospective study. Sci. Rep. 6:39189. 10.1038/srep39189 27995960 PMC5171914

[B14] Yang L. Wu D. (2021). Grit and meaning in life of Chinese nurses: the chain mediating effect of social support and hope. Front. Psychol. 12:769707. 10.3389/fpsyg.2021.769707 34858295 PMC8631816

[B15] Yang S. Huang H. Qiu T. Tian F. Gu Z. Gao X.. (2020). Psychological capital mediates the association between perceived organizational support and work engagement among Chinese doctors. Front. Public Health 8:149. 10.3389/fpubh.2020.00149 32528918 PMC7256625

